# Surface-Enhanced Raman Imaging of Intracellular Bioreduction of Chromate in *Shewanella oneidensis*

**DOI:** 10.1371/journal.pone.0016634

**Published:** 2011-02-25

**Authors:** Sandeep P. Ravindranath, Kristene L. Henne, Dorothea K. Thompson, Joseph Irudayaraj

**Affiliations:** 1 Bindley Bioscience Center, Birck Nanotechnology Center, Agricultural and Biological Engineering. Purdue University, West Lafayette, Indiana, United States of America; 2 Argonne National Laboratory, Chicago, United States of America; 3 Department of Biological Sciences, University of Tennessee, Knoxville, Tennessee, United States of America; University of California Merced, United States of America

## Abstract

This proposed research aims to use novel nanoparticle sensors and spectroscopic tools constituting surface-enhanced Raman spectroscopy (SERS) and Fluorescence Lifetime imaging (FLIM) to study intracellular chemical activities within single bioremediating microorganism. The grand challenge is to develop a mechanistic understanding of chromate reduction and localization by the remediating bacterium *Shewanella oneidensis* MR-1 by chemical and lifetime imaging. MR-1 has attracted wide interest from the research community because of its potential in reducing multiple chemical and metallic electron acceptors. While several biomolecular approaches to decode microbial reduction mechanisms exist, there is a considerable gap in the availability of sensor platforms to advance research from population-based studies to the single cell level. This study is one of the first attempts to incorporate SERS imaging to address this gap. First, we demonstrate that chromate-decorated nanoparticles can be taken up by cells using TEM and Fluorescence Lifetime imaging to confirm the internalization of gold nanoprobes. Second, we demonstrate the utility of a Raman chemical imaging platform to monitor chromate reduction and localization within single cells. Distinctive differences in Raman signatures of Cr(VI) and Cr(III) enabled their spatial identification within single cells from the Raman images. A comprehensive evaluation of toxicity and cellular interference experiments conducted revealed the inert nature of these probes and that they are non-toxic. Our results strongly suggest the existence of internal reductive machinery and that reduction occurs at specific sites within cells instead of at disperse reductive sites throughout the cell as previously reported. While chromate-decorated gold nanosensors used in this study provide an improved means for the tracking of specific chromate interactions within the cell and on the cell surface, we expect our single cell imaging tools to be extended to monitor the interaction of other toxic metal species.

## Introduction

Chromium (Cr) is an important industrial metal used in the fabrication of a wide range of products and applications including alloys, leather tanning, textile processing, electroplating, printing inks, refractories and several other industries [1]. Due to its widespread use in industry, chromate [hexavalent chromium, Cr(VI)] has become a pervasive contaminant in the environment, making it a serious public health and environmental concern [2]. Chromium (Cr) can exist in valence states ranging from −2 to +6, of which Cr(VI) and Cr(III) are the most stable forms [3]. Chromium (III) is an essential nutrient used by the human body in processing sugar, protein, and fat [4]. Hexavalent chromium [Cr(VI)], on the other hand, is a known “human carcinogen” [5] whose inhalation has been linked to lung cancer according to the International Agency for Research on Cancer. Cr(VI) is readily soluble in alkaline environments [6], posing a threat to ground water quality as it can mobilize and spread quickly. For these reasons, the U.S. EPA (Environmental Protection Agency) has designated chromium as a “priority pollutant” [7] and considerable measures have been taken to effectively remediate and safely detoxify chromium-polluted soil and aquatic environments. Bioremediation is a promising approach for cheap, effective, and rapid *in situ* remediation of polluted environments [8]. Bioremediation offers multiple advantages over competing technologies by way of *in situ* decontamination, utilization of natural processes that are specific to the target contaminant [9], and significant reduction of additional environmental stresses [10]. Although bioremediation has vast potential in dealing with intractable environmental problems, much of this promise has yet to be realized. Specifically, much needs to be learned about what drives remediating microorganisms and their interactions with their surrounding chemical and biological environment [9].

Hexavalent chromium has been linked not only to cancer, respiratory and skin irritation, but has been shown to adversely affect bacterial survival and vitality of soil microbial communities [11], [12], [13]. Chromium remediation in particular is challenging as it is present in more than half of all the U.S.-EPA designated toxic superfund sites [14]. Several microorganisms are known to be capable of reducing toxic forms of chromium, but few are capable of reducing multiple metallic and organic contaminants typically present in contaminated sites. Based on its diverse repertoire of metal reduction and metabolic capabilities [15], *Shewanella oneidensis* MR-1 is considered as a model organism for metal reduction and is a vital tool in the potential remediation of waste sites contaminated with toxic materials such as uranium, vanadium, chromium, and radionuclides [16], [17], [18]. The significance of this strain is further reflected in the formation of the “Shewanella Federation”, a large, Department of Energy (DOE)-funded collaboration that applies bioinformatic, genomic, and proteomic techniques to define the systems biology of Shewanella [16], [19], [20] in cell populations.

Bioreduction of chromate to the less soluble and less toxic trivalent chromium is one mechanism microorganisms may use to alleviate the toxicity of hexavalent chromium. However, the enzymes and molecular mechanisms involved in the bioreduction of chromate are not completely understood. In strain MR-1 alone, there is evidence of multiple reduction mechanisms [21], [22], [23]. Some bacterial strains can reduce chromate through soluble proteins [24], [25], [26], and chromate reduction has also predominantly been shown to occur at the surface of other bacteria [27], [28]. In the case of *S. oneidensis* MR-1, chromate reduction may occur within the cytoplasm as well as at the cell surface; however, the degree and location of chromate reduction appeared to be dependent on culture conditions [29]. The observations presented in the study by Middleton et al [29] relied upon identifying electron dense regions by TEM (Transmission Electron Microscopy) and electron energy loss spectroscopy to infer the cellular location of chromium. The methods used in these prior studies were rather nonspecific and showed diffuse patterns of chromium throughout the cell [29]. Chromate-decorated gold nanosensors used in this study provide an improved means for the tracking of specific chromate interactions within the cell and on the cell surface. Our research focuses on chromate reduction by *S. oneidensis* MR-1 as a means to explore and develop nanotools for mapping intracellular processes at single-cell resolution. We present a unique approach of using chromate-gold nanosensors to detect the cellular chromate reduction sites in *Shewanella oneidensis* MR-1. Inert chromate-gold nanoparticle sensors were fabricated [30] and used as probes to monitor metal reduction processes in single cells. Finally, we demonstrate a Raman chemical imaging strategy utilizing nanoparticles as a first step towards exploring the intracellular reduction of environmentally toxic metals such as chromate by metal-reducing bacteria.

The proposed single cell investigation constitutes the first step toward defining a pathway to map metal reduction sites to better understand the mechanisms involved in the reduction of Cr(VI) to Cr(III). Our targeted approach with Cr-decorated gold nanoparticles allows for a more definitive analysis of Cr localization and speciation within Cr-reducing cells, with the potential for adaptation to other toxic metal species. The ability to map processes through the use of sensitive spectroscopic tools is a significant step and complements existing molecular approaches by defining not only what processes are occurring within a cell, but where they are occurring at single-cell resolution. Nanoparticles have predominantly been used for biosensing and in biomedicine to detect biomolecular targets [31], [32]. The present work integrates surface-enhanced Raman spectroscopy (SERS) with multifunctional nanomaterial to monitor biomolecular interactions in bacterial cells, and points to a more fundamental application of nanotechnology in understanding cellular processes. SERS, a surface sensitive technique, generates large amplifications of the laser field driven by local plasmon resonances resulting in enhancement of Raman scattering of molecules that are either attached to metal surfaces or held at close proximity [33]. Owing to its single molecule sensitivity [34] and inherent molecular specificity, SERS has been very effective in delivering valuable chemical fingerprint information in biological systems [35], [36]. Several SERS substrates have been developed for whole organism fingerprinting of bacteria useful for detection and identification of several strains of bacteria [36]. While numerous studies have been able to get intracellular SERS in mammalian cells, there are considerably fewer reports on Intracellular SERS in bacteria [37]. To our knowledge, this is the first report of the detection of chromate reduction and chromium speciation using SERS imaging.

## Results and Discussion

### Fabrication and Characterization of chromate-coated gold nanoprobes: Cr-AuNp

**Figure 1:**
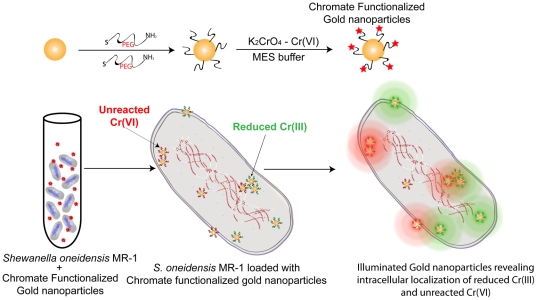
Schematic Illustration. Representation of passive uptake of Cr-AuNPs by *S. oneidensis* MR-1 and subsequent Raman Chemical imaging of cells to reveal the intracellular localization of reduced Cr(III) and unreacted Cr(VI).

As illustrated in Figure 1, in order to track the cellular sites of reductive reactions, chromate-coated gold nanoparticles (Cr-AuNps) 3.5 and 13 nm in diameter [38] were fabricated. The negatively charged gold nanospheres were first stabilized with multifunctional PEG (Polyethylene glycol) molecules (SH-PEG-NH_2_), which have dual functionality with gold-binding thiol groups as well as surface accessible amine groups.These probes were first incubated in MES (Morpholineethanesulfonic acid) buffer to activate the amine groups, followed by prolonged interaction with chromate molecules to form a uniform layer of chromate on the nanoparticle surfaces. The electrostatic interaction between chromate (oxyanion) and amine groups on the particles form a strong linkage to hold chromate onto the surface of the particles. Cr-AuNps were found to be highly stable based on their zetapotential values (∼32 mV), indicative of the strength of the colloidal suspension. Ultimately, the Cr-AuNp probes were purified using multiple centrifugation steps to remove free chromate in the suspension before the probes could be used for cellular studies.

### Evaluation of the effect of Cr-AuNP on growth and chromate reduction by *S. oneidensis* MR-1

**Figure 2:**
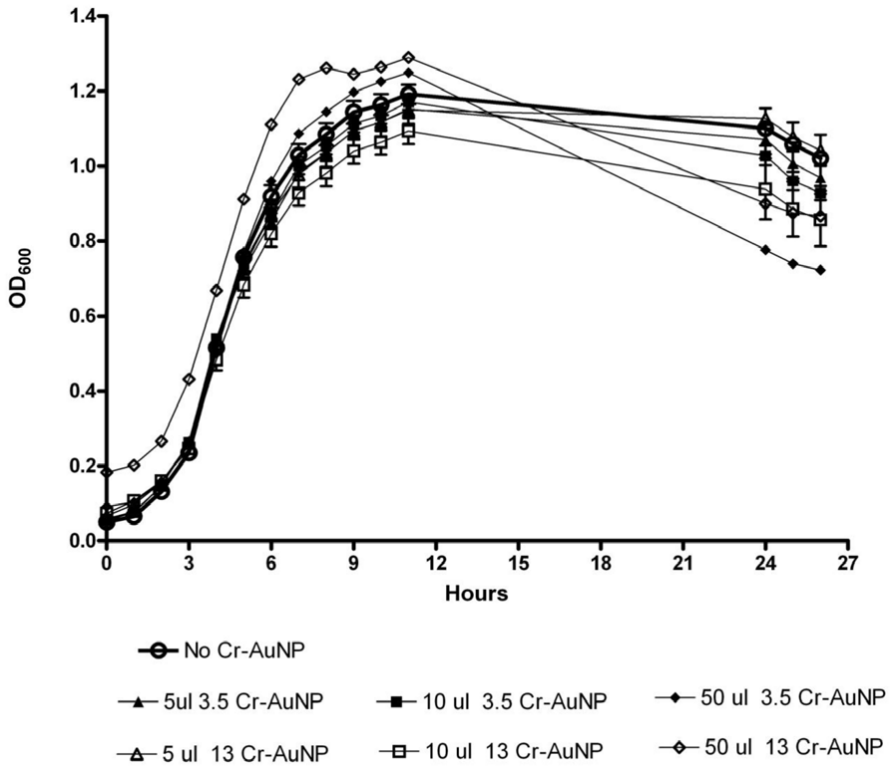
Effect of Cr-AuNPs on the growth of *Shewanella oneidensis* MR-1. Either 3.5 nm (closed symbols) or 13 nm (open symbols) Cr-AuNPs were added to wells at volumes of 0, 5, 10, and 50 µl. Error bars represent the standard error from three independent cultures.

A detailed investigation of cell viability was conducted to ensure that gold nanoparticles and the chromate-coated nanoprobes are not toxic to cells as shown in Figure 2. Either 3.5 nm (closed symbols) or 13 nm (open symbols) Cr-AuNPs were added to the wells at volumes of 0, 5, 10, and 50 µl. There was no apparent adverse effect on growth induced by either size Cr-AuNP in the logarithmic phase, and each culture was able to attain similar maximal OD_600_. Additional volumes of Cr-AuNPs yielded similar results (data not shown).

**Figure 3:**
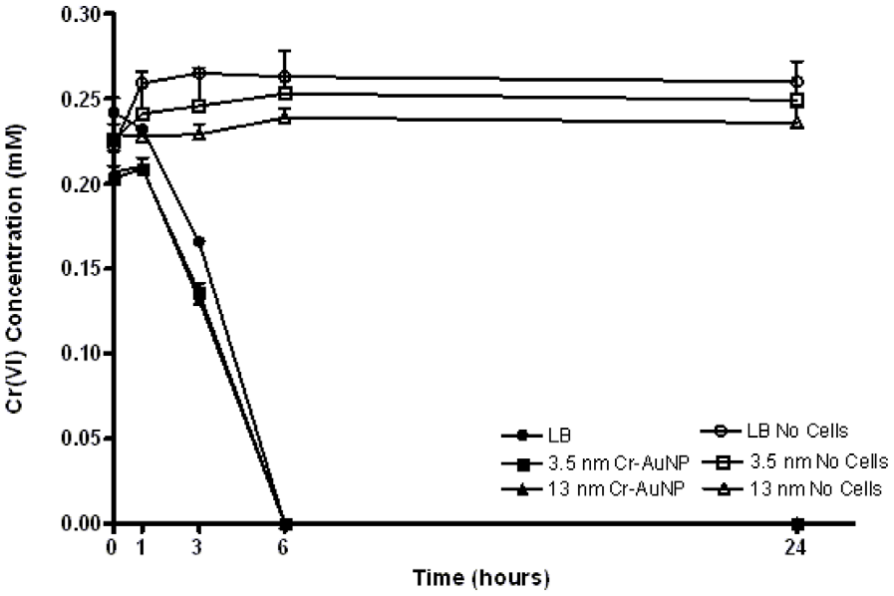
Effect of Cr-AuNPs on chromate reduction by *S. oneidensis* MR-1. Abiotic controls were included to rule out the reduction of chromate by media components and the Cr-AuNPs (open symbols). There was no adverse effect on chromate reduction ability induced by the nanoparticles (closed symbols). In addition, the nanoparticles did not directly reduce the chromate in the medium in the absence of cells (open symbols). Error bars represent the standard error from three independent cultures.

In probing for cellular sites of Cr(VI) reduction, it was of utmost importance to maintain the normal cellular physiology and growth of the microorganisms. To monitor chromate reduction by *S. oneidensis* MR-1, bacteria were incubated with Cr-AuNp in a passive manner in the absence of any applied external force (such as electroporation). This method enabled us to study the parameters of chromate uptake by cells and to track Cr-AuNp localization sites as depicted in the illustration (Figure 1). In the presence of a range of dilutions of Cr-AuNps, there was no difference in the ability of *S. oneidensis* MR-1 to reduce chromate compared to the nanoprobe-free control (Figure 3). There was no adverse effect on chromate reduction ability induced by the nanoparticles (closed symbols). In addition, the nanoparticles did not directly reduce chromate in the medium (open symbols). This ensures that the reductive probing occurred under biomimetic chromate-reducing conditions.

### Passive uptake: Incubation with nanoprobes

**Figure 4:**
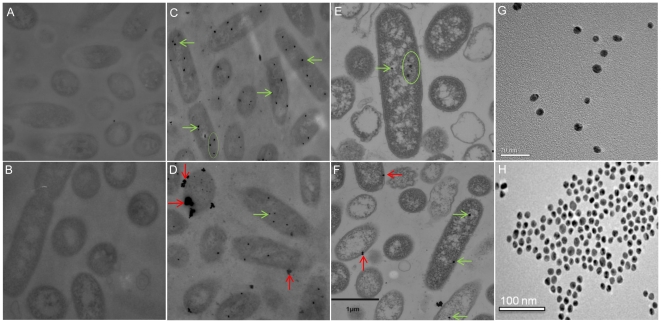
Thin-section TEM Images of *S. oneidensis* MR-1. A. without particles, B. plain 13 nm gold Nanoparticles, Fig. 4C–4D. Chromate coated gold nanoparticles, Cr-AuNp:13 nm, (Fig. 4E–4F) 3.5 nm Cr-AuNp. Red arrows indicate extracellularly bound Cr-AuNp and green arrows/circle indicate internalized particles. (Fig. 4G–4H) show 3.5 nm and 13 nm probes used in Cr-AuNp preparation.

Unlike mammalian cells, bacteria lack the endocytosis mechanisms to uptake nanoparticles or biomolecules [39]. When *S. oneidensis* cells were incubated with untreated gold nanoparticles (3.5 nm/13 nm) in LB broth (13 nm shown in Figure 4b), the organisms did not internalize particles irrespective of the colloidal concentration and the incubation time. When the particles were functionalized with thiolated-PEG (SH-PEG, MW 5000), an increase in stability of the probes was observed, but there was no noticeable change in their uptake (data not shown). In addition, no uptake of these particles was observed when incubated in the presence of chromate in the incubating matrix (data not shown). However, a drastic improvement in the uptake (internalization) of the particles by cells was noted when Cr-AuNps were used as shown in Figure 4c–f. *S. oneidensis* MR-1 internalized both 3.5 nm and 13 nm gold nanoparticles when decorated with chromate (K_2_CrO_4_). In addition to 3.5 nm and 13 nm nanoprobes, we also attempted uptake experiments with 40 nm sized chromate coated probes. However, the bacterial uptake achieved with 40 nm probes was considerably lower than that attained with the use of 3.5 nm or 13 nm particles. While we have not characterized the transport mechanism responsible for this uptake, it is very likely that the Cr-conjugated gold nanoparticles are taken up through sulfate transport systems. It is accepted that bacterial chromate uptake occurs through sulfate transporters due to the structural similarity of chromate and sulfate ions. Competitive inhibition of sulfate uptake by chromate has also been demonstrated [40], [41]; however, internalization could possibly occur through dedicated chromate receptors that are yet to be characterized. Multiple levels of interaction ranging from mere physical binding to the cell surface to complete internalization of Cr-AuNp probes into the bacterial cells could occur. Since the cells were thoroughly washed prior to imaging, an almost complete removal of non-specifically bound probes is assured.

### Confocal Raman spectral evaluation

**Figure 5:**
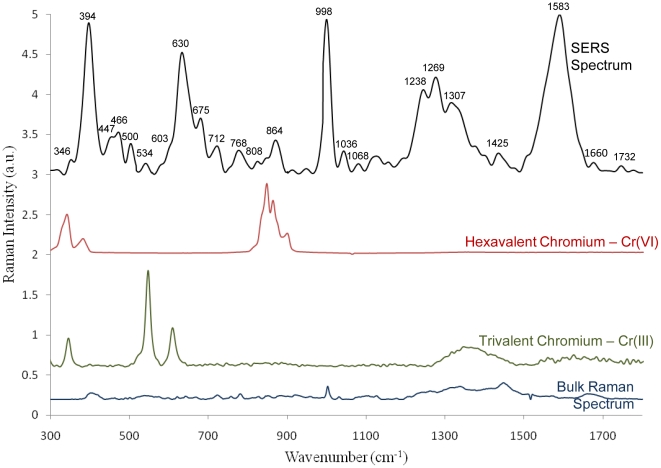
Raman Spectral Analysis. Single cell SERS spectra obtained from *S. oneidensis* MR-1 incubated with 3.5 nm gold nanoparticles depicting the uptake of gold nanoparticles by this organism. chromium(VI) peaks are highlighted with red and the reduced chromium(III) peaks are marked in green. Chromium(VI) –red and Chromium (III) – green spectra are aligned to show their peak positions in bacteria. Bulk Raman spectrum acquired from a cluster of chromate reducing cells is also shown.

The SENTERRA confocal Raman system (Bruker Optics, Billerica, MA) fitted with a 785 nm laser, and a 100× air objective (N.A. 0.7) was used to study *S. oneidensis* cells associated with Cr-AuNp. An integration time of 10–20 s and a laser power of 10 mW at the laser source were used to investigate the band shift due to Cr-AuNp localization within the cell. Even though the control samples (*S. oneidensis* without any gold nanoparticles) gave rise to Raman spectra, the signal at these measurement parameters was not sufficiently enhanced without gold nanoparticles. Raman signal was observed from sites containing gold nanoparticles in the vicinity. A Raman peak at 200–260 cm^−1^ due to the presence of gold nanoparticles in the focal volume was observed. This low-frequency Raman peak is indicative of the plasmon-phonon coupling effect resulting from acoustic vibrations of surface characteristics selected by the resonant excitation of localized plasmons [42]. The presence of this peak indicates the presence of chromate-coated gold nanoparticles in the focal volume. TEM images (Figure 4) clearly show the presence of aggregates rather than, or in addition to, individual particles. Figure 5 illustrates a sample spectrum obtained from *S. oneidensis* MR-1 with passively internalized gold nanoparticles providing the necessary enhancement for intracellular Raman mapping. Samples were optimized for Raman mapping based on spectral acquisition and analysis on single cells.

Raman spectra of potassium chromate (K_2_CrO_4_) were obtained at the same experimental settings. Three major modes of vibrations were observed in the Raman spectra of Cr(VI) at 341, 387, and 846 cm^−1^. Raman spectra for Cr(III) (chromium oxide: Cr_2_O_3_) revealed four sharp Raman bands at 281, 332, 545, and 606 cm^−1^. This distinct difference in the Raman spectra of these two forms of chromium has enabled us to speciate intracellular chromium reduced by *S. oneidensis* MR-1. The major peaks relevant to Cr(VI) and Cr(III) were identified in Cr-AuNp loaded cells. A differential detection of Cr peaks in some cells were observed showing peaks of either Cr(VI) or Cr(III), while other cells did not show any Cr peak. This points to the fact that the cells within a bacterial culture may co-exist in a range of physiological states and do not reduce metal to the same extent. Such single-cell interrogation helps to better assess the reductive state of individual cells in a colony so that effective reduction capacities can be estimated. With the appropriate integration of cell sorting tools, organisms with superior reductive capacities can be isolated for designing effective bioremediation strategies.

### Raman Chemical Imaging

**Figure 6:**
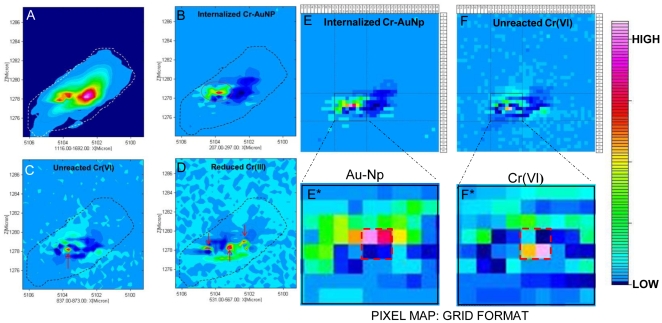
Confocal Raman Mapping. Raman Intensity Maps averaged over a wide wavenumber region (162–1953 cm^−1^) covering most of bio-molecular components in cells to obtain a Raman chemical image of the cell (A), Phonon Plasmon peak (207–297 cm^−1^) originating from gold depicting the presence of Cr-AuNps (B), Cr(VI) - hexavalent chromium (C, 837–873 cm^−1^), reduced non-toxic trivalent Cr(III) (D, 531–567 cm^−1^). Raman images in grid format, Fig. 6E and 6F are representations of 6B and 6C respectively. 6-E* and F* represent magnified pixel plots to demonstrate the overlap in signal of Au and Cr(VI) peaks within cells.

Different levels of interaction between the cells and the chromate-decorated nanoparticles were noted. While some cells showed only the phonon-plasmon peak without revealing any biomolecular signatures, more cells revealed crucial vibrational signatures of cellular constituents, implying the close association of chromate coated nanoparticles with cells. When the interaction between the cells and the probes is high, characteristic peaks relevant to that of chromate as well as lipid/phospolipid or membrane protein peaks were more obvious. Raman peaks relevant to nucleic acids were also observed. It was important to make use of this signal specificity to identify chromate reduction sites within the cell. Confocal Raman mapping in single cells show the distribution of Cr-AuNp within the cell as well as the spread of Cr(VI) and Cr(III) molecules. A laser setting of 25 mW with 10 s integration time was used to obtain a 4×6 µm^2^ area Raman image by constructing a 40 by 40 grid. The resulting Raman image is a plot of signal intensity of a specific wavenumber region generated from the acquisition of 1600 spectra over the 4×6 µm^2^ spatial grid constructed in this particular case. Since the spatial resolution of the instrument with the 100× objective is ∼1 µm^2^, a considerable overlap in the signal collection was necessary; however, spectral processing was accomplished to remove the overlapping region. The integrated contour plots for specific peak positions of the resulting Raman maps are shown in Figure 6(a, b, c & d). Raman maps for the cells were generated by integrating specific peak intensity over the mapped area. A broad wavenumber region 162–1953 cm^−1^ encompassing most of the cellular constituents were obtained to create a Raman chemical image of the cell (a). The dashed line boundary depicts the periphery of the cell. When the phonon-plasmon peak region 207–297 cm^−1^ (b) was integrated, the position of gold nanoparticles within the cell could be located. Similarly, integration of the signal intensity over the 837–873 cm^−1^ and 531–567 cm^−1^ revealed intracellular localization of Cr(VI) and Cr(III), respectively. While the contour plots revealed a considerable spread of gold nanoparticles throughout the cell, there is a considerable overlap of Cr(VI) with gold nanoparticles. The individual pixel-based grid map shown in Figure 4 accurately tracks the respective overlap into a narrow four pixel region of an area of approximately 60 nm^2^. While this is slightly larger than the area occupied by one Cr-AuNp, indicating that the signal obtained might originate from a cluster or aggregates of the probes resulting in a SERS hot spot. This spatial overlap in signal helps us to confirm not only the cellular internalization of gold nanoprobes, but also the oxidative state of chromate coated on the internalized probes. It is to be noted that the chromate was functionalized to gold nanoparticles based on the electrostatic interaction between the positive amine groups and the negatively charge oxyanionic CrO_4_^2−^. However, when the cells reduce Cr(VI) to Cr(III), cationic Cr(III) can no longer bind directly to gold probes due to electrostatic repulsion with the positively charged gold probes. Nevertheless, we still see Cr(III) signal within cells as shown in Figure 6-d which matched well with the plasmon-phonon peak depicting the presence of gold nanoparticles which acts as a SERS substrate to enhance the signal of Cr(III) within the effective SERS range that is most effective within 1–10 nm [43], [44] of the metal surface and decays exponentially, reaching 40% of the enhancement maximum when the distance between the target and the SERS substrate is ∼30 nm [45]. Thus, by Raman chemical imaging, we identify chromate reduction pockets in a bacterium.

### Fluorescence Lifetime single cell Imaging

**Figure 7:**
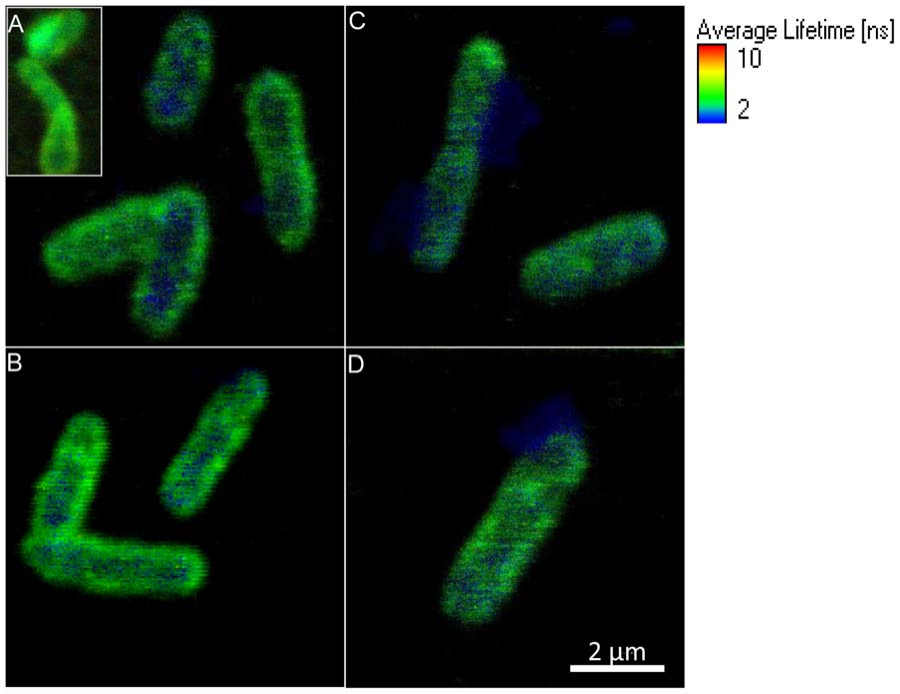
Confocal Fluorescence Lifetime Imaging. *S. oneidensis* MR-1 incubated with 3.5 nm (Fig. 7A & 7C) and 13 nm (Fig. 7B & 7D) Cr-AuNp probes show scattered low-lifetime (blue) distribution indicating the presence of gold nanoparticles (both internalized and externally bound) compared to the control incubated with plain gold nanoparticles (inset - 7A).

Confocal Fluorecence Lifetime Images were obtained using Green Fluorescence Protein (GFP) expressing *S. oneidensis* MR-1 incubated with Cr-AuNp under pre-determined conditions (Figure 7- a, b c & d) to validate the Raman chemical images. Fluorescence lifetime images were obtained from the lifetime of each pixel as determined by *τ_D_* (fluorescence lifetime). Fluorescence lifetime imaging (FLIM) permits the visualization of contrast between materials at different fluorescence decay rates present in a confocal volume. The average lifetime of the peaks from FLIM is characteristic of the exponential decay of the fluorophore. As observed in Figure 7-a,c (3.5 nm Cr-AuNP), there is an uneven distribution of fluorescence lifetime (lifetime of GFP is ∼2.1 ns) in single cells compared to the control (with plain AuNP) that has an even distribution of GFP. In the confocal volume, when the cells are associated with gold, which has a very low lifetime (∼0.4 ns) decay, the average lifetime within the pixel reduced significantly, leading to a scattered bluish green lifetime distribution within the cell. Similar results were observed within cells incubated with 13 nm particles. FLIM imaging is effective in mapping the regions associated with gold within cells. In some samples, it was easy to determine the localization of the gold nanoparticles either within or at its periphery (Figure 7-c,d).

### Inductively Coupled Plasma Mass Spectrometry

**Figure 8:**
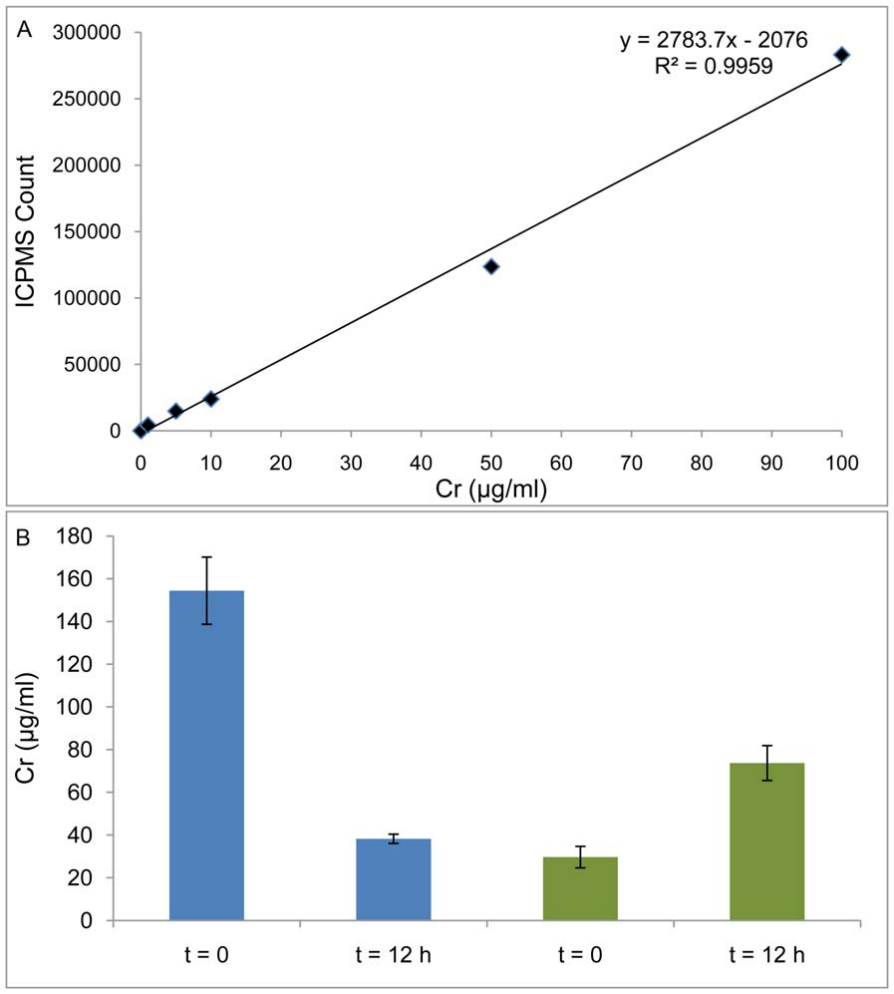
Inductively Coupled Mass-Spectrometry. A. ICP-MS Calibration curve for Cr quantification. B. Intracellularly trapped Cr(VI) and Cr(III) at time t = 0 and t = 12 h after Cr-AuNp treatment.

Further confirmation studies to quantitate intracellularly trapped Cr(VI) and Cr(III) as well as validate their presence within cells were performed by Inductively Coupled Plasma Mass-Spectrometry (ICP-MS) in cell populations. A dynamic reaction cell (DRC) mediated sample chamber was employed to eliminate polyatomic interferences (^40^Ar^12^C^+^, ^1^H^35^Cl^16^O^+^, ^40^Ar^13^C^+^ and ^37^Cl^16^O^+^) and an amino-propyl coated silica based ion-exchange column was used to achieve Chromate speciation [46]. The calibration curve for Cr quantification is shown in Figure 8a. Figure 8b indicates the presence of intracellular Cr(VI) and Cr(III) both at the beginning of the reaction and after an incubation period of 12 h. We can observe that at t = 0, the intracellular Cr(VI) is higher than Cr(III) and after prolonged incubation, the intracellular Cr(VI) quantities decrease, while Cr(III) values show an increase.

In this work, we have developed and demonstrated a chemical imaging platform to monitor chromate reduction in *S. oneidensis* MR-1 at the single-cell level using chromate-coated gold nanoparticles. Cr-AuNp internalization was confirmed with thin-section TEM and Fluorescent lifetime imaging. Bacterial SERS spectra were acquired and characterized for chromate reduction. Single-cell Raman chemical imaging was optimized and used for studying intracellular chromate reduction to identify both forms of chromium at nanometer-scale resolution. The spectral and mapping information confirms specific intracellular chromate reduction and localization sites within cells. The ease of probe preparation and subsequent imaging provides an effective means to circumvent the use of synchrotron based X-ray techniques. SERS and FLIM-based approaches could be used to further characterize chromate and other metal reduction sites and rates in live *S. oneidensis* MR-1 exposed to environmentally relevant conditions.

## Materials and Methods

### Fabrication of Cr-AuNp- 3.5 nm/13 nm

Spherical gold nanoparticles of sizes 3.5 nm and 13 nm were used as the primary substrates for chemical functionalization. 13 nm sized gold nanoparticles were synthesized using the standard citrate mediated reduction of HAuCl_4_. 3.5 nm gold nanoparticles were synthesized based on the NaBH_4_ activated citrate mediated reduction of HAuCl_4_ to obtain citrate capped 3.5 nm stable nanocolloids [38]. The surfaces of these bare nanoparticles are negatively charged, which were functionalized with multifunctional PEG molecules containing a thiol group (-SH) at one end and an amino group (-NH_2_) on the other. Thiolated end of the PEG molecules can bind to surfaces of gold nanoparticles with amino groups exposed to the solvent. These amino groups were activated in MES buffer before incubation with K_2_CrO_4_ solution for 24 h to form Cr(VI) coated gold nanoprobes.

### Toxicity studies by monitoring growth curves

Growth of *Shewanella oneidensis* MR-1 was followed through stationary phase in the presence of increasing concentrations of chromate conjugated gold nanospheres (Cr-AuNPs) to determine their effects on bacterial growth. An overnight culture of strain MR-1 in Luria Broth (LB) diluted to an approximate optical density (Optical Density- OD_600_) of 0.02 was used as a starting culture and distributed into 200 ml aliquots in a 96-well plate. Either 3.5 or 13 nm Cr-AuNPs were added to wells at volumes of 0, 5, 10, 20, 30, 40, 50 and 100 µl. All assays were performed in triplicates. Turbidity measured at 600 nm periodically on a Perkin Elmer 1420 microplate reader was plotted.

### Monitoring Chromate reduction by *S. oneidensis* MR-1

The chromate starter cultures with O.D_600_∼0.2 from an overnight culture of MR-1 were challenged with 0.3 mM K_2_CrO_4_ to monitor the effect of Cr-AuNp on bacterial chromate reduction. Cr-AuNps were added to the above cultures in three concentrations: 0, 0.1 ml (Low), 0.25 ml (Medium) and 0.5 ml (High). Abiotic control sets with similar concentrations of Cr-AuNps were prepared. Cultures constituted samples obtained at 0, 1, 3, 6 and 24 hours for Cr(VI) concentration using the ChromaVer-3 (Diphenylcarbazide, DPC) assay [47].

### Confocal Raman Imaging and spectroscopic measurements

A SENTERRA confocal Raman system (Bruker Optics, Billerica, MA) fitted with a 785 nm laser, and a 100× air objective (N.A. 0.7) was used to study *S. oneidensis* cells associated with Cr-AuNp. An integration time of 1–20 s and a laser power of 1–25 mW at the laser source was used to investigate the band shift due to Cr-AuNp localization within the cell. A specific z-plane was selected for confocal imaging. A 6×6 µm^2^ grid (area) was chosen around the target cell, and the number of grids was selected anywhere between 100–1600 resulting in a 10×10 to 40×40 grid on the cell and a relevant step size in nanometer range with individual grid size of ∼0.02 µm^2^.

### Construction of GFP and YFP-expressing *Shewanella oneidensis* MR-1 strains

Plasmids p18 and p19 carrying genes for EGFP and YFP (Yellow Fluorescent Protein) expression, respectively, were obtained from the laboratory of Jennifer Morrell-Falvey of Oak Ridge National Laboratory. Electrocompetent *S. oneidensis* MR-1 cells were obtained by washing 100 ml of mid-logarithmic phase cells three times in sterile, chilled distilled water and once in sterile, chilled 10% glycerol. The cell pellet was resupsended in 500 µl 10% glycerol. Electrocompetent cells were quickly frozen on dry ice and stored at −80°C. Approximately 20 ng of plasmids p18-EGFP and p19-YFP were mixed with 300 µl electrocompetent cells. Transformation was carried out using the Nucleofector II device (Amaxa, Inc., Koeln, Germany) with bacterial setting AG. Transformants were selected on Luria Agar plates supplemented with 10 µg/ml gentamycin.

### Confocal Fluorescence Lifetime Imaging Instrumentation

Fluorescence Lifetime measurements were performed on a time-resolved scanning confocal microscope (Microtime 200, Picoquant GmbH, Berlin, Germany); 465 nm pulse laser was used to excite the GFP-expressing *S. oneidensis* MR-1. The laser beam was focused onto the sample through an apochromatic 60×, 1.2 NA water immersion objective, and the emitted fluorescence was acquired using the same objective. The excitation beam was subtracted using a dual band dichroic (z467/638rpc, Chroma). A 50 µm pinhole was used to reject the off-focus photons from the excitation volume, and the overall fluorescence was collected and separated accordingly using a dichroic beam splitter (600 dcxr, AHF, Chroma) and filtered by emission filters before being detected by two single photon avalanche photodiodes (SPAD) (SPCM-AQR, PerkinElmer Inc.).

### ICP-MS/Chromate Quantification

Inductively Coupled Plasma – Mass Spectrometer (ICP-MS) measurements were done using a quadrupole ELAN DRC-e spectrometer (PerkinElmer SCIEX, Ontario, Canada), integrated with a Dynamic Reaction Cell (DRC) to quantify the initial concentration of Cr(VI) and the reduced Cr(III). The sample delivery system consisted of a PerkinElmer auto sampler model AS-93 Plus tray, peristaltic pump and a cross-flow nebulizer with a double pass spray chamber. The same instrumental conditions were used for quantification of both Chromium Cr(VI) and Cr(III). Chromabond NH_2_/3 mL/500 mg columns (i.e. Aminopropyl phase with a 3 mL volume and 500 mg of sorbent) were obtained from Macherey-Nagel (Duren, Germany). These columns were activated and used for speciation of Cr(III) from Cr(VI) both in the cells and the supernatant [46].
